# The multisensory mind: a systematic review of multisensory integration processing in Anorexia and Bulimia Nervosa

**DOI:** 10.1186/s40337-023-00930-9

**Published:** 2023-11-16

**Authors:** Giulia Brizzi, Maria Sansoni, Daniele Di Lernia, Fabio Frisone, Cosimo Tuena, Giuseppe Riva

**Affiliations:** 1 Applied Technology for Neuro‐ Psychology Laboratory, IRCCS Istituto Auxologico Italiano, Via Magnasco 2, 20149 Milan, Italy; 2 Humane Technology Laboratory, Università Cattolica del Sacro Cuore, Largo Gemelli, 1, 20121 Milan, Italy; 3 Department of Psychology, Università Cattolica del Sacro Cuore, Largo Gemelli, 1, 20121 Milan, Italy

**Keywords:** Anorexia, Bulimia, Multisensory integration, Crossmodal integration, Body experience, Allocentric lock

## Abstract

Individuals with Anorexia Nervosa and Bulimia Nervosa present alterations in the way they experience their bodies. Body experience results from a multisensory integration process in which information from different sensory domains and spatial reference frames is combined into a coherent percept. Given the critical role of the body in the onset and maintenance of both Anorexia Nervosa and Bulimia Nervosa, we conducted a systematic review to examine multisensory integration abilities of individuals affected by these two conditions and investigate whether they exhibit impairments in crossmodal integration. We searched for studies evaluating crossmodal integration in individuals with a current diagnosis of Anorexia Nervosa and Bulimia Nervosa as compared to healthy individuals from both behavioral and neurobiological perspectives. A search of PubMed, PsycINFO, and Web of Sciences databases was performed to extract relevant articles. Of the 2348 studies retrieved, 911 were unique articles. After the screening, 13 articles were included. Studies revealed multisensory integration abnormalities in patients affected by Anorexia Nervosa; only one included individuals with Bulimia Nervosa and observed less severe impairments compared to healthy controls. Overall, results seemed to support the presence of multisensory deficits in Anorexia Nervosa, especially when integrating interoceptive and exteroceptive information. We proposed the Predictive Coding framework for understanding our findings and suggested future lines of investigation.

## Abstract (plain language summary)

Diagnoses of Anorexia Nervosa and Bulimia Nervosa have increased dramatically in recent years, taking on the characteristics of a mental health emergency. More research is therefore needed to better understand these conditions, especially given their complex and multifaceted nature. Patients affected by these conditions report significant alterations in body-self experience. Body experience results from a cross-modal integration process in which information from different sensory modalities and spatial frames is combined. Therefore, we systematically reviewed studies that focused on multisensory integration in patients affected by Anorexia Nervosa and Bulimia Nervosa, as it may play a key role in the onset and maintenance of these pathologies. Studies in this review found multisensory integration difficulties in patients with Anorexia Nervosa, but not enough studies were retrieved to draw conclusions regarding Bulimia Nervosa. We discussed findings trying to link behavioral, neuropsychological, and neuroscientific evidence in light of the predictive coding framework to provide a different perspective on patients' distorted body experiences. This may lead to new insights to refine our understanding of these complex and poorly understood disorders.

## Introduction

Eating disorders (EDs) are mental illnesses characterized by abnormal eating behaviors and distorted thoughts about food, weight, and body shape. The National Institute of Mental Health in 2021 estimates that approximately 9% of the population experiences EDs, highlighting these pathologies may deserve special attention. In particular, the number of diagnoses of Anorexia Nervosa and Bulimia Nervosa has increased dramatically in recent years [1], taking on the characteristics of a mental health emergency. Indeed, these conditions are associated with serious consequences for the well-being of the individual [2], and high mortality rates [3].

According to the Diagnostic and Statistical Manual of Mental Disorders Fifth Edition (DSM-5; [4]), Anorexia Nervosa (AN) is characterized by a significant reduction in food intake, resulting in extremely low body weight and an overwhelming fear of becoming fat. Specifically, individuals affected by AN experience distorted perceptions of their body image and weight, which is closely associated with the adoption and maintenance of pathological behaviors such as severe food restriction and/or bingeing in an attempt to control body weight. Similarly, individuals affected by Bulimia Nervosa (BN) manifest body representation disturbances, in addition to altered eating behaviors characterized by recurrent binge eating episodes followed by compensatory strategies (e.g., laxative abuse, self-induced vomiting, or fasting) to regulate body weight [5]. Then, despite substantial differences, Anorexia Nervosa and Bulimia Nervosa symptomatology present similarities in the way patients experience their bodies [6].

Our team's findings from two 4-years longitudinal studies, one with 2713 female college students [7] and the other with 2507 male college students [8] support the critical role of the body in the development of Eating Disorder symptomatology. In both groups, the onset and persistence of DSM-5 EDs at the 4-years follow-up were predicted by self-objectification, body dissatisfaction, internalization of the appearance ideal, dieting, and negative affectivity at baseline, as well as changes in these factors. Although all of these vulnerability factors are known to play a key role in the development and maintenance of EDs (see, for example, [9, 10]), these studies revealed that different factors have distinct predictive values. First, body dissatisfaction and internalization of appearance ideals explain almost twice as much variance in the onset of symptoms as dieting and negative emotions. In addition, self-objectification, which is the tendency to adopt a third-person perspective on the self—i.e., to see one's body from the perspective of an outsider as an object to be viewed and evaluated based on its appearance [11]—explained almost four times more variance of EDs symptoms than dieting and over four times more than negative affects.

Particularly, when individuals objectify themselves, they tend to internalize an objectified self-image by using an allocentric frame of reference (observer mode) to recall events in which they judge themselves mainly based on how they look [12]. A large number of studies supported the critical role of self-objectification in weight and eating disorders, stressing how it directly links the experience of the body to an individual's identity and values.

Thus, data from clinical practice and research agree that the body plays an important role in the development of Eating Disorders. As a result, a deeper understanding of body experience is necessary to fully understand how to appropriately support patients with EDs.

### The Body in Eating Disorders

Body experience is a multidimensional construct that includes feelings, cognitions, and perceptions about one’s body [13, 14]. The attitudinal component refers to experienced feelings about one’s body appearance, the cognitive component consists of beliefs and attitudes about one’s body, while the perceptual component refers to subjective expectations about one’s body (e.g., recognizing, estimating, and identifying the own body size and shape; [15]). Research has consistently shown that patients with AN and BN are characterized by body alterations at all of these levels [15, 16].

Several reviews and meta-analyses have highlighted abnormalities in the way individuals affected by AN and BN experience their bodies from both a behavioral and neural perspective, emphasizing changes in affective and cognitive components (e.g., high levels of body dissatisfaction and body concerns; [17, 18]). The presence of perceptual deficits instead remains still an open debate. A large body of research has shown that people with AN and BN tend to misperceive their body size (e.g., they overestimate their body dimensions [19, 20]). Several studies investigated possible mechanisms involved in determining this phenomenon. Most studies have examined visual impairments (e.g., abnormal visual adaptation or visual scanning) as a possible explanation for the misperception, but they yielded inconsistent results [20]. These discrepancies may be related to the impossibility of reducing a complex phenomenon such as bodily experience to the purely visual domain [21]. Indeed, the body is considered one of the most complex perceptual objects [22, 23]. To fully understand this statement, it is necessary to understand where bodily perception comes from and what its underlying mechanisms are.

### The Body and Multisensory Integration

The body is a special perceptual object compared to others because it requires the processing and merging of information coming from both outside and inside the body itself, namely exteroceptive (e.g., visual, somatosensory, auditory signals relayed from the outside world by sense organs), interoceptive (e.g., visceral and vestibular signals originating from sensory nerve endings), and proprioceptive (e.g., the sense of body position originating from muscles and joints) signals respectively [14].

In this view, our experience of the body results from the combination of a continuous stream of different information from the body to the brain, in a process defined as Multisensory Integration (MSI; [22]). Going into detail, MSI refers to a sophisticated mechanism by which inputs from two or more senses (e.g., visual, tactile, auditory) are combined to enhance each other, resulting in a final product that cannot be directly disassembled to recover the components from which it was formed [23, 24]. To successfully merge signals, the brain must identify which of them contain related information—i.e., solve the correspondence problem—integrate this information and dynamically resolve spatial or temporal conflicts that may arise between sensory modalities [25].

In particular, for this combination to occur, the signals must be mapped to a common spatial frame. However, unimodal sensory areas have the disadvantage of encoding spatial positions differently in each modality, which poses a challenge when considering how multisensory representations are generated [26]. The term “spatial frames” refers to the reference frame from which a sensory signal is derived: specifically, it is possible to distinguish between egocentric (i.e., first-person perspective) and allocentric (i.e., third-person perspective) frames [27]. Avillac et al. [28] suggested that unimodal reference frames are not remapped into a common reference frame, but rather adapt to the dominant modality: that is, signals from different sensory domains are aligned to the reference frame of the leading sense modality.

**Fig. 1:**
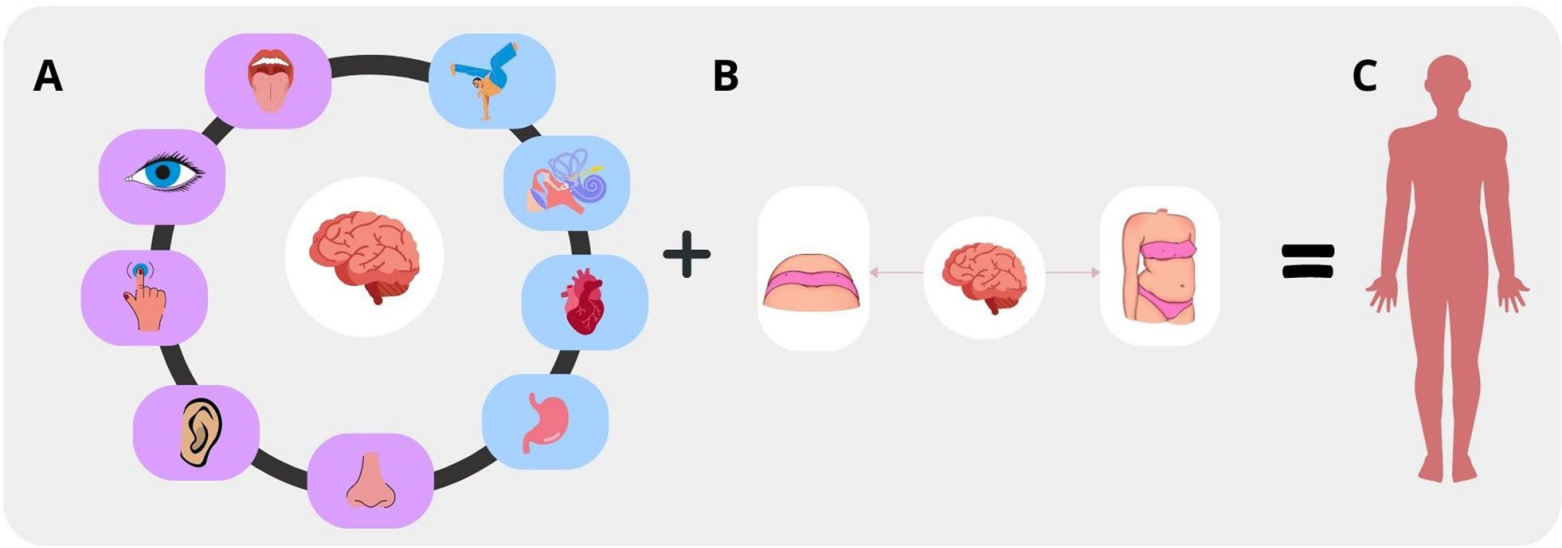
Multisensory Integration Processing in its two main components. Panel A shows different sensory modalities, including exteroceptive information (purple) and inner body signals (i.e., proprioceptive, vestibular, and interoceptive; blue); Panel B shows an example of information from egocentric (left) and allocentric (right) spatial frames; in the example we refer to visual information from first and third-person perspectives. Panel C shows the output of the coherent integration of all this information, namely the body matrix

Putting all of these steps together, how we experience our bodies results from the integration of sensory information from both different modalities and from the allocentric and egocentric spatial frames into a single and coherent percept (Fig. 1). The result of this process is a supramodal body representation called “body matrix” [14]. In other words, we can conceptualize bodily experience as a jigsaw puzzle, where different elements are put together through a computational process so that the final perceptions appear seamless.

Neurological and neuropsychological studies have revealed the crossmodal and fragmentary nature of bodily experience not only in neurological conditions (e.g., spatial neglect, phantom limb) but also in healthy individuals thanks to experimental manipulations exploiting multisensory conflicts [29]. A classic example is the Rubber Hand Illusion (RHI; [30]), in which the spatially and temporally synchronous occurrence of multimodal visual-tactile stimulation applied over rubber and the real hands of participants leads individuals to experience a fake hand as their real hand, thus altering their bodily experience. Following the same principles, advanced multisensory technologies—such as Virtual Reality (VR)—have been used to create cross-modal conflicts (e.g., visual and tactile conflicts) that induce individuals to embody a full virtual body (Full Body Illusion; FBI; [31]). Paradigms such as the RHI and FBI are examples of the critical role of MSI in shaping and determining how we make experience of our bodies.

### The Body, Multisensory Integration, and Eating Disorders

How people experience their bodies plays a key role in the development and maintenance of Anorexia Nervosa and Bulimia Nervosa, and research revealed that body experience is the result of a multisensory integration process. Therefore, a possible relationship between EDs and multisensory integration abilities was hypothesized. Specifically, it has been proposed that impairments in multimodal integration abilities may contribute to altered body experience (e.g., [15, 32]). A growing body of research from the fields of cognitive science, neuroscience, and neuropsychology appears to support this hypothesis. Preliminary studies have shown that patients with AN and BN show abnormalities in the integration of stimuli from different sensory modalities (e.g., audio-visual integration deficits during the Sound-Induced Flash Illusion; [33]), and revealed cortical abnormalities in brain regions hypothesized to be involved in cross-modal integration (e.g., frontal and parietal regions; [34]).

Rephrasing altered body experience in these terms, symptoms related to body misperception might reflect a deeper deficit of multisensory integration abilities rather than a malfunction of a single sensory modality (i.e., the overestimation of one's weight would not result from a mere visual deficit, but from the inability to combine crossmodal information necessary for the construction of one's body representation; [35, 36]).

Reading Eating Disorders through crossmodal integration concept could provide an important building block for achieving a better understanding of these conditions. This is crucial in light of the increasing number of AN and BN diagnoses and the limited effectiveness of current interventions (e.g., cognitive behavioral therapy, and family-based treatment; [37]). Such approaches mostly focus on the cognitive, behavioral, and interpersonal functioning of patients, leaving the bodily experience in the background. They are motivated by the idea that working on higher-level (top-down) processes may have indirect benefits on the way patients experience their bodies. However, this approach may be necessary but not sufficient: given the key role of the body, it may also be useful to understand and work from a perceptual (bottom-up) perspective.

Thus, the study of the multisensory integration process could provide important insights into the development of intervention protocols that can work from both a rational (top-down) and intuitive (bottom-up) perspective and address different aspects of patient functioning. This follows Bruch’s [38] suggestion, according to which relevant improvements in Eating Disorder pathologies are likely to be temporary remissions without addressing how patients experience their bodies.

Despite evidence suggesting that multisensory integration may play a critical role in AN and BN [21], to our knowledge no study has reviewed the empirical evidence on this topic. Therefore, the present systematic review aimed to investigate MSI in individuals with AN and BN to see whether these conditions are characterized by alterations in this process. Thus, we reviewed studies that assessed the behavioral dimension of MSI or its neurobiological underpinnings to obtain a complete picture of the phenomenon. We argue that this review process could support the analysis of possible gaps within this literature as well as suggest possible areas of investigation for further research to deepen understanding of EDs.

## Methods

### Protocol and registration

A systematic review of scientific literature was performed to identify studies assessing MSI in individuals with AN and BN. The present review was carried out following the Preferred Reporting Items for Systematic Reviews and Meta-Analyses (PRISMA; [39]) and it was pre-registered in the PROSPERO register (CRD42022383008).

### Search strategy

Data sources were collected on December 12, 2022, through a computerized search of three prominent databases: PubMed, PsycINFO, and Web Of Science. Each was searched independently using a specific search string consisting of terms with different variations and truncations indicating the population and outcome of interest. The string below was used to filter the titles, abstracts, and keywords of the articles:

anorexia OR bulimia OR "eating disorder*" AND.

multisensory OR "sensory integration" OR multimodal OR allocentric OR egocentric OR "frame of reference" OR “touch” OR “proprioception” OR “visuo-tactile” OR “auditory”

Multisensory and frame-of-reference-related terms were used with the OR operator according to Avillac's conceptualization of multisensory integration [26], while sensory modality terms were selected according to Stein et al. [23] definition of MSI. We also used terms referring to single sensory modalities to broaden the search, although we included “visuo-tactile” instead of “visual” because much research outside the area of interest has been conducted to assess unimodal visual abilities in EDs. We did not a priori define a specific starting year of publication for the articles to be included. Details of each search result are provided in Table 1 to allow for future replication of the study. The complete list from each database was imported into Rayyan [40] to detect duplicates.

**Table 1:** Search strategy

Anorexia AND	PubMed	Web Of Science	PsycINFO
Multisensory	21	20	19
“Sensory integration”	2	2	14
Multimodal	135	141	118
Allocentric	9	9	12
Egocentric	9	10	12
“Frame of reference”	4	4	8
Touch	34	81	61
Proprioception	9	18	9
Visuo-tactile	4	9	2
Auditory	54	69	63
Bulimia AND			
Multisensory	5	4	4
"Sensory integration"	1	1	3
Multimodal	25	36	72
Allocentric	3	3	2
Egocentric	4	4	4
"Frame of reference"	1	1	3
Touch	7	40	21
Proprioception	2	4	3
Visuo-tactile	0	2	0
Auditory	8	20	22
Eating Disorder* AND			
Multisensory	30	25	37
"Sensory integration"	4	6	31
Multimodal	110	108	206
Allocentric	14	14	21
Egocentric	17	17	23
"Frame of reference"	4	4	25
Touch	28	30	105
Proprioception	4	7	17
Visuo-tactile	1	5	4
Auditory	36	33	115
Sub Total	585	727	1036
Total			2348
Duplicates removal			1437
Identified studies for screening			911
Included studies			13

Description of search strategy; the number of studies for each string in the included database, the total number of studies before and after duplicate removal, and the final studies included in the review are reported.

### Eligibility criteria

We screened titles, abstracts, and full texts of the 911 individualized studies for inclusion criteria after eliminating duplicates. Specifically, we included studies that:

- Were available in English;
- Included were samples of adolescents and/or adults with a diagnosis of AN or BN based on the DSM—specifically the Fourth (DSM IV and DSM-IV-TR) and Fifth (DSM-5) editions—or the International Statistical Classification of Diseases and Related Health Problems, Tenth Edition (ICD-10), as these are the most recent and widely used diagnostic tools. Thus, studies using other diagnostic manuals or earlier editions of the DSM or ICD were excluded; we also excluded studies that included AN and BN patients in the same group (patients with AN and BN were grouped, referred to as the "ED group") or that did not separate the two conditions in the data analysis. We did not consider the presence of possible confounding factors (i.e., comorbidities) as an exclusion criterion because of the limited number of studies in this area. In addition, studies that focused on non-clinical or subclinical populations (e.g., individuals with disordered eating), as well as other clinical conditions outside of those of interest (e.g., obesity, binge eating disorder) were excluded; studies involving animals were also not included. Notably, we chose to focus on AN and BN because of their similarities in terms of how patients experience their bodies, and in light of evidence reporting diagnostic crossover between these two conditions [41];
- Included a control group of healthy participants (HCs), a group of individuals without a diagnosis of EDs or any other pathological condition; when studies included participants with Anorexia Nervosa, we included only those studies in which the average body mass index (BMI) in the healthy control group was higher than that of the clinical group;
- Assessed MSI by using behavioral tasks (e.g., Rubber Hand Illusion) or neuroimaging techniques (e.g., functional magnetic resonance imaging);
- Conceptualized MSI according to the definitions of Stein et al. [23] and Avillac et al. [26, 28]. As a consequence, we included behavioral studies that: used MSI paradigms in which at least two different sensory modalities were stimulated at the same time (e.g., aperture task, size-weight illusion, sound-induced flash illusion), used multisensory technologies (e.g., Virtual Reality) to stimulate multiple sensory modalities at a time, or/and manipulated the spatial frame of reference (i.e., allocentric and egocentric; e.g., [42]) in which the task is proposed. Regarding neuroscience studies, we included studies that analyze MSI brain correlates in the populations of interest during multisensory tasks. Accordingly, studies investigating one sensory modality at a time and studies using resting-state neuroimaging techniques were not included. Additionally, studies investigating only bodiless imagery tasks (e.g., motor imagery task; [43]) were not included;
- Were research studies evaluating MSI skills in individuals with AN and/or BN. Thus, other types of publications—such as editorials, book chapters, conference proceedings, reviews, and meta-analyses—were excluded; only peer-reviewed original articles were included;
- Were either quantitative behavioral studies or neuroscience research. Then, qualitative studies were not included.

## Results

**Fig. 2:**
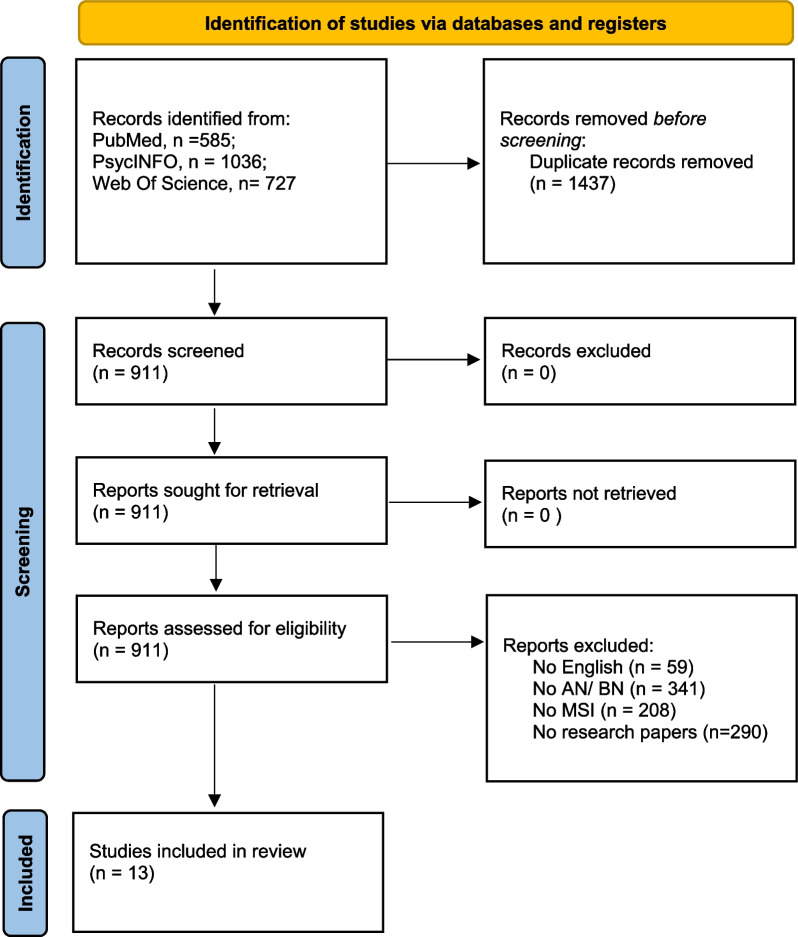
PRISMA flowchart. Flowchart presenting the extraction and selection process to reach the final number of included studies

Of 2348 studies retrieved from PubMed, Web Of Science, and PsycINFO databases, 911 were unique articles. We screened the titles, abstracts, and full texts of these studies, ultimately including 13 articles in this review. The remaining studies were excluded because they did not meet the inclusion criteria. The most common reasons for rejection were: (a) studies did not include the population of interest (e.g., obese patients, non-clinical conditions, diagnosis not based on the specified diagnostic manuals), (b) they used multisensory paradigms and tasks to modify eating disorder symptoms or as a treatment, (c) they did not include a control condition [44], (d) they were other types of publications (e.g., editorials, reviews, meta-analyses, book chapters, and (e) they did not assess multisensory integration abilities as conceptualized here (e.g., they used neuroimaging techniques without multisensory tasks; e.g., [45]). Further details of the study selection process are provided in the PRISMA flowchart (Fig. 2).

### Quality assessment

Two reviewers (G.B., M.S.) separately assessed the risk of bias according to the checklist for assessing methodological quality proposed by Downs and Black [46]. This tool allows the evaluation of the methodological quality of both randomized and nonrandomized comparative studies across relevant parameters: specifically, reporting strategy, external and internal validity as well as power. Examples of items are “Are the characteristics of the patients included in the study clearly described?”, “Were the subjects asked to participate in the study representative of the entire population from which they were recruited?”, and “Was an attempt made to blind those measuring the main outcomes of the intervention?”[46]. Disagreements and/or ambiguities were resolved by consensus. The results of the risk of bias assessment are presented in Table 2. All included studies were considered to be fair or good in terms of bias according to the scoring criteria.

**Table 2:** Risk bias assessment

Item from the Downs and Black checklist	Beckmann et al. 2021	Case et al. 2012	Chirico et al. 2019	Eshkevari et al. 2012	Keizer et al. 2016	Keizer et al. 2019	Guardia et al. 2010	Guardia et al. 2012	Guardia et al. 2013	Metral et al. 2014	Provenzano et al. 2019	Risso et al. 2020	Zopf et al. 2016
Item 1	1	1	1	1	1	1	1	1	1	1	1	1	1
Item 2	1	1	1	1	1	1	1	1	1	1	1	1	1
Item 3	1	0	0	1	1	1	1	1	1	1	0	1	1
Item 4	1	0	1	1	1	1	1	1	1	1	1	0	1
Item 5	1	0	0	1	1	1	1	1	1	1	0	1	0
Item 6	1	0	1	1	1	1	1	1	1	1	1	0	1
Item 7	1	1	1	1	1	1	1	1	1	1	1	1	1
Item 8	0	1	0	0	0	1	0	0	1	0	0	0	0
Item 9	0	1	1	1	0	1	1	1	1	0	0	0	0
Item 10	1	1	1	1	1	1	1	1	1	1	1	1	1
Item 11	1	1	1	1	1	0	1	1	1	1	1	1	1
Item 12	1	1	1	1	1	0	1	1	1	1	1	1	1
Item 13	1	1	1	1	1	1	1	1	1	1	1	1	1
Item 14	0	0	0	0	0	1	0	0	0	0	0	0	0
Item 15	0	0	0	0	0	0	0	0	0	0	0	0	0
Item 16	1	1	1	1	1	1	1	1	1	1	1	1	1
Item 17	0	0	0	0	0	1	0	0	0	0	0	0	0
Item 18	1	1	1	1	1	1	1	1	1	1	1	1	1
Item 19	1	1	1	1	1	1	1	1	1	1	1	1	1
Item 20	1	1	1	1	1	1	1	1	1	1	1	1	1
Item 21	1	1	1	1	1	1	1	1	1	1	1	1	1
Item 22	1	1	1	1	1	1	1	1	1	1	1	1	1
Item 23	0	0	0	0	0	1	0	0	0	0	0	0	0
Item 24	0	0	0	0	0	1	0	0	0	0	0	0	0
Item 25	1	0	0	0	1	1	1	1	1	1	0	0	1
Item 26	0	1	1	1	1	1	1	1	1	1	1	1	1
Item 27	1	0	0	0	0	1	0	0	0	0	0	0	0
TOTAL SCORE	**19**	*16*	*17*	**19**	**19**	**24**	**20**	**20**	**21**	**19**	*16*	*16*	*18*

Risk of bias assessment was performed following Downs and Black [46] assessment tool for items. Please confront the original tool for items explanation; note: 0 = No/Unable to determine, 1 = Yes. Only question 5 could be answered 0 = No, 1 = partially, 2 = Yes, Cut-off total score: 24–28 points (excellent), 19–23 points (good; bold values), 14–18 points (fair; italic values), 14 points or less (poor)

### Classification criteria

As introduced earlier, how we perceive our body results from the integration of information from different sensory modalities and spatial reference frames (i.e., multisensory integration).

Among the different sensory domains, we can distinguish between exteroceptive modalities—which include visual and tactile information- and interoceptive modalities—which include interoceptive, proprioceptive, and vestibular information. Some modalities -such as haptic information- are hybrid since they require co-participation of information from both the external environment and within the body.

Based on this distinction, we classified the studies according to the sensory modalities stimulated by the tasks used [47]. Based on previous research [15] tactile input was considered present when the task involved a stimulus that touched the participants' skin. We distinguished it from haptic input, which instead refers to conditions where the task requires active haptic exploration of things (e.g., touching/grasping an object; [48]. We considered proprioceptive input to be present when the task required judgments about body parts and/or overall body posture, and vestibular ones where tasks required moving and/or maintaining specific body orientation [49]. Interoceptive input was considered to be present when the task tested sensitivity to visceral activity [15]. Finally, visual input was considered to be present if the task required viewing the body-related stimuli (i.e., the whole body and/or specific body parts, virtual bodies) or if the task involved visual stimuli to which participants had to respond. The frame of reference was classified according to the spatial perspective in which the tasks were presented (i.e., first- and/or third-person perspective).

Regarding the different spatial frames, we reported studies that went on to manipulate the spatial perspective in which the specific task was presented. In particular, this criterion fits well with tasks such as body illusions, as they can be presented from an egocentric (first-person) or/and allocentric (third-person) perspective.

### Data extraction

In the following sections, study characteristics and main results are presented to address the research question, namely the investigation of MSI abilities in individuals with AN and BN. As we did not find any neuroimaging studies that met our inclusion criteria, only behavioral studies will be presented. Detailed study characteristics, including relevant sample characteristics—such as age, body mass index (BMI), disease duration, gender, and diagnosis—sensory modalities involved, paradigms, techniques used to assess MSI, and main results for each study are summarized in Table 3. The spatial frame of reference was specified in the paradigms section where applicable. In addition, the interpretation of the main results according to the authors of the studies is also presented.

**Table 3:** Studies characteristics

Study	Sensory modalities involved	Paradigms and measures	Main outcomes	Main outcomes interpretation	Sample features (diagnosis, subtype, size, mean age, mean BMI, mean illness duration, and relative SD/ SE)	Gender
Provenzano et al. (2021)	Visual–Tactile–Proprioceptive	Full Body Illusion in Virtual Reality from egocentric (1st person) perspective) Embodiment measures: Embodiment questionnaire (see [50]), skin temperature Emotional Valence: Visual Analogue Scale Avatar Selection Task in Virtual Reality from allocentric (3rd person) perspective	*Full Body Illusion in Virtual reality Embodiment measures* No significant differences between groups in embodiment measures *Emotional Valence* Patients with AN experienced more negative emotions after being exposed to the avatar with a greater BMI as compared to the avatar with a lower BMI. The opposite pattern was instead found in the control group *Avatar Selection Task in Virtual Reality* Individuals with AN and HCs differed in body dissatisfaction, with patients reporting overall higher levels of body dissatisfaction and a desire to have a thinner body No differences were observed between groups in the real body estimation task; however, participants in both groups tended to overestimate their body dimensions	Individuals with Anorexia Nervosa might present alterations in the affective and emotional components of body experience, rather than in the perceptual one	AN (Subtype restrictive) = 20; age (years) = 23.30 ± 7.61 years; BMI (kg/m^2^) = 15.87 ± 1.12; illness duration NR; diagnostic criteria DSM-5 HCs = 20; age (years) 23.85 ± 3.23; BMI (kg/m^2^) = 18.98 ± 1.01	F
Case et al. (2012)	Visual-Haptic-Proprioceptive Integration	Size Weight Illusion [51]	Patients with AN experienced the illusion (and the reversed size weight illusion) significantly less than controls. This is despite normal weight discrimination ability	Individuals with AN seem to present abnormal visual-haptic-proprioceptive sensory integration abilities which might explain the reason why the visual perception of the body in a mirror does not correct the patient's distorted body representation image	AN (Subtype NR) = 10; age (years) = 29.1 ± 11.0 years; BMI (kg/m^2^) = 17.1 ± 0.9; illness duration (months) = 36 (SD NR); diagnostic criteria DSM-IV-TR HCs = 10; mean age 25.8 ± 9.0 years; mean BMI 21.7 ± 1.6 kg/m^2^	F
Zopf et al. (2016)	Visual—Tactile—Proprioceptive	Rubber Hand Illusion from egocentric (1st person) perspective [30] Embodiment measures: Embodiment questionnaire [30, 32] Body perception measures: Reaching task [52]	*Rubber Hand Illusion and Embodiment Questionnaire* Patients with AN reported significantly higher embodiment scores than controls in synchronous conditions *Body perception measures* Differences in reaching trajectories were found between the groups; specifically, individuals with AN made more reach-to-grasp endpoint errors toward external visual information compared to controls	Individuals affected by AN show deficits in processing visual-proprioceptive signals compared to controls. Thus, the results seem to support the idea that multisensory perception of body position is abnormal in individuals with AN	AN (Subtype NR) = 23; age (years) = 21.87, SD = 2.79; BMI (kg/m^2^) = 15.82, SD = 1.27; illness duration (years) = 5.26, SD = 3.60; diagnostic criteria DSM-IV HCs = 23; age (years) = 21.48, SD = 2.35; BMI (kg/m^2^) = 21.16, SD = 2.10	F
Guardia et al. (2013)	Visual–Haptic–Proprioceptive–Vestibular	Spatial orientation constancy and body orientation perception: Subjective Vertical Task and body Z– axis perception[53]	*Subjective Vertical Task* Patients with AN showed a higher A-effect—meaning a deviation of the tactile and visual subjective verticality towards the body under tilted conditions *Body Z-axis* No differences emerged between groups concerning tactile and visual body Z-axis judgments in the upright position; differences instead emerged when tilting the body, where individuals with AN judged the body as more tilted compared to controls	Participants with AN seem to present multisensory deficits in spatial orientation which can relate to a decrease in somatosensory information processing; such difference could be explained in terms of	AN (Subtype restrictive) = 25; age (years) = 22.24, SD = 8.59 years; BMI (kg/m^2^) = 14.89, SD = 1.10; illness duration (years) = 4.57, SD = 6.52; diagnostic criteria DSM- IV-TR HCs = 25; age (years) = 22.88, SD = 3.63; BMI (kg/m^2^) = 21.65, SD = 1.72	F
Risso et al. (2020)	Visual-Tactile	Multisensory Processing Assessment task [54]	Individuals with AN tended to overestimate the width of the ellipse more than HCs in the tactile modality, whereas no differences were observed in the visual condition Participants with AN showed a higher visual and tactile discrimination threshold compared to controls Individuals with AN accurately integrated tactile and visual information as HCs; however, differences emerged in terms of discrimination thresholds: the clinical group showed a lower bimodal threshold as compared to unimodal conditions whereas HCs showed similar thresholds across conditions	Patients with AN might have difficulties in visual and tactile sensory processing instead of deficits in multisensory integration	AN (Subtype restrictive) = 19; age (years) = 26.12, SD = 9.34; BMI (kg/m^2^) = 15.15, SD = 2.64; illness duration NR; diagnostic criteria DSM-5 BCHC = 9; age (years) = 25.67, SD = 2.18; BMI (kg/m^2^) = 20.49, SD = 1.43 HCs = 19; age (years) = 24.47, SD = 1.26; BMI (kg/m^2^) = 19.86, SD = 1.94	F
Beckmann et al. (2020)	Visual-Proprioceptive- Vestibular	Aperture task from egocentric (1st person) perspective [49]	Patients with AN reported a higher critical aperture/ shoulder ratio than HCs, meaning that patients rotate their shoulders for relatively larger door widths compared to controls	Individuals with AN tend to rely on an incoherent body schema to plan and perform actions; specifically, they tend to unconsciously estimate their body size to be larger than in reality	AN (Subtype restrictive) = 18; AN (Subtype binge/purge) = 5; age (years) = 24.67, SD = 5.61; BMI (kg/m^2^) = 14.60, SD = 1.88; illness duration (years) = 5.81 SD = 4.83; diagnostic criteria ICD-10 HCs = 21; age (years) = 24.19, SD = 3.12; BMI (kg/m^2^) = 21.95, SD = 1.30	F
Chirico et al. (2019)	Auditory-visual	Sound-Induced Flash Illusion [55]	Patients with AN were less accurate compared to HCs for each SOA and sensory modality No significant differences were observed between groups in the visual condition for SOA from 70 to 110 ms; however, individuals with AN showed difficulties in visual trials with longer SOAs (i.e.,150 ms, 230 ms) Participants with AN were never able to detect the double sound in the auditory condition In the visual condition, the clinical group reported a higher number of correct responses compared to the other two modalities; difficulties emerged for longer visual SOA, as well as for the bimodal condition across all SOA	AN showed an impaired ability to integrate auditory and visual stimuli	AN (Sybtype NR) = 9; age (years) = 30, SD = 10.46; BMI (kg/m^2^) = 15.59, SD = 1.96; illness duration NR; diagnostic criteria DSM-5 HCs = 9; age (years) = 24.56, SD = 1.67; BMI (kg/m^2^) = 22.69, SD = 2.14	F
Eshkevcari et al. (2012)	Visual—Tactile—Proprioceptive	Rubber Hand Illusion from egocentric (1st person) perspective Embodiment measures: Embodiment Questionnaire, Proprioceptive drift Finger localization task	*Embodiment Questionnaire* Clinical groups composed of patients with AN and BN reported higher levels of embodiment than HCs However, these differences were not significant when controlling for mood *Proprioceptive drift* Patients with AN showed higher proprioceptive drift compared to HCs, whereas participants with BN did not differ from controls *Finger localization task* Individuals with AN showed more biased finger location estimation compared to controls, whereas patients with BN did not differ from HCs	People with eating disorders show a plastic bodily self compared to healthy controls. This malleability may stem from alterations and deficits in processing body-related information	AN (Sybtype restrictive) = 24; AN (Subtype binge/purge) = 12; age (years) = 23, SD = 18; BMI (kg/m^2^) = 16.1, SD = 2.71; illness duration (years) = 6, SD = 11; diagnostic criteria DSM-IV-TR BN = 22; age (years) = 22.5, SD = 10; BMI (kg/m^2^) = 20.9, SD = 4.28; illness duration (years) = 7, SD = 4; diagnostic criteria DSM-IV-TR ENDOS = 20; age (years) = 27.5, SD = 16; BMI (kg/m^2^) = 19.7, SD = 5.54; illness duration (years) = 11.5, SD = 12; diagnostic criteria DSM-IV-TR HCs = 61; age (years) = 24.7, SD = 7; BMI (kg/m^2^) = 21.5, SD = 2.80	F
Keizer et al. 2016	Visual–Tactile–Proprioceptive	Full Body Illusion in Virtual Reality from egocentric (1st person) perspective Embodiment measures: Embodiment Questionnaire Body Size Estimation Task	*Embodiment Questionnaire* No significant differences were observed between groups concerning illusion strength as reflected by Embodiment Questionnaire *Body Size Estimation Task* Patients with AN tended to misestimate their body width and circumferences more than controls	Participants with AN may base their body size on the most recent visual information available to them. The tendency of people with AN to overestimate their body size is not the result of a general bias to perceive their own body as larger than reality, but it is specifically limited to perceiving the body as wider and rounder	AN (Subtype NR) = 30; age (years) = 22.03, SD = 3.67; BMI (kg/m^2^) = 18.11, SD = 1.68; illness duration (months) = 110.61, SD = 11.62; diagnostic criteria DSM-IV HCs = 29; age (years) = 21.07, SD = 2.34; BMI (kg/m^2^) = 20.77, SD = 1.48	F
Keizer et al. 2019	Visual-Proprioceptive- Vestibular	Hoop Task from egocentric (1st person) perspective	At the baseline, participants affected by AN tended to misestimate the smallest hoop that would allow them to fit through compared to controls	Patients with AN seem to present a suboptimal ability to derive body size estimates from multisensory information processing of bodily information	AN (subtype NR) = 12; age (years) = 23.17, SD = 5.67; BMI (kg/m^2^) = 20.11, SD = 1.17; illness duration (months) = 12.29, SD = 10.32; diagnostic criteria DSM-5 AN (subtype NR) = 14; age (years) = 22.87, SD = 2.90; BMI (kg/m^2^) = 19.53, SD = 1.04; illness duration (months) = 7, SD = 3.93; diagnostic criteria DSM-5 HCs = 20 age (years) = 21.21, SD = 1.44; BMI (kg/m2) = 20.80, SD = 1.61	F
Metral et al. 2014	Visual-Proprioceptive- Vestibular	Aperture Task from egocentric (1st person) perspective asking both to image and pass through doorway-like apertures	Individuals with AN showed a higher passability ratio (i.e. the ratio between the critical aperture size and shoulder width) relative to HCs, both in motor imagery and real action aperture tasks	Patients with AN show body schema alterations that influence action planning. Moreover, such alteration seems to resist corrections stemming from sensorimotor information generated when performing motor tasks. These results suggest that the central nervous system in individuals affected by this condition might be locked to a false representation of the body that cannot be updated	AN (2 subtype binge/purge, 12 rescriting) = 14 age (years) = 24.14, SD = 0.65; BMI (kg/m^2^) = 14.70, SD = 1.50; illness duration (years) = 5.71, SD = 9.67; diagnostic criteria DSM-IV-R HCs = 14; age (years) = 25.21, SD = 7.77; BMI (kg/m^2^) = 21.62, SD = 1.82	F
Guardia et al. 2010	Visual- Proprioceptive- Vestibular	Aperture Task from egocentric (1st person) perspective	The group composed of individuals with AN showed an abnormally higher critical aperture size to shoulder width ratio compared to controls. Additionally, this misestimation correlated with the duration of illness and the degree of body concerns	Body size overestimation consistently observed in individuals with AN might not merely be due to psychological and affective factors; specifically perceptual processing of the body at a neural level might be impaired (i.e., alterations at the level of the parietal network)	AN (subtype NR) = 25; age (years) = 24.32, SD = 6.54 BMI (kg/m2) = 15.14, SD = 1.55; illness duration (years) = 5.3, SD = 4.8; diagnostic crtieria DSM IV HCs = 25; age (years) = 23.04, SD = 5.98; BMI (kg/m2) = 21, SD = 1.99	F
Guardia et al. 2012	Visual-Proprioceptive- Vestibular	Aperture Task from both egocentric (1st person) and allocentric (3rd person) perspectives	Individuals with AN showed a higher passability ratio compared to HCs when the task was proposed from egocentric but not from allocentric spatial reference frame	The body overestimation and the alteration in body schema reliably observed in individuals with AN might reflect a deeper deficit in the crossmodal integration ability of bodily information	AN (12 subtype restricting, 13 binge/purge subtype = 25; age (years) = 28.84, SD = 7.75; BMI (kg/m^2^) = 15.65, SD = 1.24; illness duration NR; diagnostic criteria DSM-IV-R HCs = 25; age (years) = 24.48, SD = 6.7; BMI (kg/m^2^) = 22.06, SD = 2.37	F

The table presents the studies' characteristics based on extraction parameters, sample features, and main findings. Abbreviations: SD = standard deviation; NR = no reported; AN = anorexic patients; BN = patients with Bulimia Nervosa; HCs = healthy controls; BMI = Body Mass Index; SOA = stimulus onset asynchrony; F = female; BCHC = body shape concerns healthy controls; ENDOS = eating disorder not otherwise specified

### Study characteristics

Table 3 shows study characteristics according to the extraction parameters.

Thirteen studies assessed multisensory integration abilities in patients with Anorexia Nervosa from a behavioral perspective [33, 42, 48, 52, 56–64]. Only one study included participants with Bulimia [57]. In addition, one study also included healthy individuals with high body concerns (BCHC; [64]) and one study included patients with eating disorders not otherwise specified (ENDOS; [57]). Only two studies manipulated the frame of reference [42, 63] proposing tasks from allocentric and egocentric spatial frames.

Included studies used MSI tasks to compare performance between patients with a current diagnosis of AN and/or BN to HCs. Diagnoses for EDs were achieved according to the DSM-5 [33, 60, 61, 63, 64], DSM-IV-TR [42, 48, 57, 59, 62], or DSM-IV [52, 58, 61] diagnostic criteria. One study used the ICD-10 [56].

Sample features were similar in terms of BMI and mean age across studies, whereas illness duration and subtype information were not always reported [33, 42, 48, 52, 60]. Notably, all studies focused on female participants.

We did not find any studies investigating the functional neurobiological basis of crossmodal integration processing under the conditions of interest. In particular, most studies used resting-state and neuroimaging techniques to investigate brain changes in AN and/or BN that might be related to MSI processing, without combining neuroimaging techniques with multisensory tasks.

Among the included studies, four studies investigated Visual–Tactile–Proprioceptive integration [52, 57, 61, 63], one investigated Visual–Haptic–Proprioceptive–Vestibular integration [59], one study focused on Visual–Tactile integration [64], one on Visual–Haptic–Proprioceptive Integration [48], and one Auditory–Visual integration [33]. The remaining five studies instead investigated Visual–Proprioceptive–Vestibular integration [42, 56, 58, 60, 62].

In the following sections, the main results of the included studies are reported and discussed according to the sensory modalities involved in the experimental tasks.

#### Visual–proprioceptive–vestibular–integration

Five studies included in this systematic review investigated Visual**–**Proprioceptive**–**Vestibular**–**Integration [42, 56, 58, 60, 62]. All of them examined individuals with Anorexia Nervosa and healthy controls when performing body-scaled action tasks. Specifically, four studies used the Aperture Task [42, 56, 59, 62] and one study used the Hoop Task [60].

The Aperture Task [49] is a behavioral task in which participants must determine whether they can fit through various door-like openings without turning their hips or shoulders. It is considered an implicit measure of body experience; specifically, it assesses body schema, namely the unconscious body representation dedicated to movement and action performance [65]. The underlying idea is that the person needs to recall their body size and compare environmental and bodily measures to evaluate whether it is possible to pass through an aperture [56]. For this process to be effective, the integration of different bodily sensory modalities (i.e., visual, proprioceptive, vestibular) is required. Thus, deficits in passability judgments and appropriately interacting with the environment reveal MSI deficits and altered body experience [56]. In this task, the outcome of interest is the ratio of participants' shoulder width to the minimum aperture width at which participants began to rotate their body, a measure called the critical aperture-to-shoulder ratio (cA/S).

All studies in this review that used this procedure found that patients with AN reported a higher passability ratio compared to HCs, meaning that they rotated their shoulders for relatively larger door widths compared to controls. Notably, cA/S was not found to correlate significantly with relevant clinical parameters such as BMI and disease duration [56]. The study by Guardia et al. [42] proposed the same task from an egocentric and allocentric perspective: in the first condition, participants had to judge their own passability through the different apertures, whereas, in the second condition, they had to estimate the passability of another person. They found that participants with AN were able to accurately judge the passability of others (i.e., allocentric perspective), similar to controls so that differences emerged only in the egocentric condition.

Only one study used the Hoop Task [60]. Here participants had to judge the smallest hula hoop they could step through. Similar to findings from the Aperture Task, individuals with AN tended to overestimate the smallest opening they could pass through.

None of the included studies investigated visual, proprioceptive, and vestibular integration in patients affected by Bulimia Nervosa.

#### Visual–tactile–proprioceptive integration

Four studies in this review [52, 57, 61, 63] examined the integration of visual, tactile, and proprioceptive bodily signals in patients with Anorexia Nervosa. This process has been examined employing body illusions: precisely, the RHI [30] and the Full Body Illusion (FBI; [66]).

The Rubber Hand Illusion [30] is a perceptual illusion in which individuals perceive a fake hand as their hand after synchronous visual-tactile stimulation of the rubber and real hands; the rubber hand is placed in the biological position of the real hand, which is instead outside the subject's visual field; then both the rubber hand and the subject's real hand are stimulated at the same time and in the same position (visual-tactile synchrony), causing the subject to experience the fake hand as the real one [30]. The underlying idea is that the embodiment of the rubber hand results from the resolution of a Visual–Tactile–Proprioceptive conflict [52]. Notably, the illusion is reduced when the multisensory visual-tactile over the real and fake bodies are not spatially and temporally synchronized (visual-tactile asynchrony; [52]. The strength of the illusion is measured by self-report measures such as the Embodiment Questionnaire [50], behavioral measures such as changes in perceived hand localization (e.g., proprioceptive drift and endpoint errors, meaning reaching responses towards visual targets), and physiological measures such as skin temperature [63].

When administering the Rubber Hand Illusion to a sample of patients with Anorexia Nervosa, Zopf et al. [52] and Eshkevari et al. [57] found patients experiencing higher embodiment levels over the fake hand compared to controls. Both the research groups found that individuals with AN experienced higher levels of body ownership over the fake hand as measured by the Embodiment Questionnaire. The same pattern was observed when endpoint errors and proprioceptive drift were considered behavioral measures of illusion strength: patients exhibited more endpoint errors and proprioceptive drift after synchronous stimulation compared to healthy controls. Notably, the same pattern was observed regardless of the condition (i.e., whether the visuo-tactile stimulation was synchronous or asynchronous). Additionally, Eshkevari et al. [57] found no between-group differences in embodiment levels as measured by the questionnaire when controlling for mood, while differences in proprioceptive drift remained. Notably, the same group reported that patients also tended to misperceive the location of their hand (i.e., closer to the midline) during the baseline hand localization tasks compared to controls.

The study by Eshkevari et al. [57] is the only study in this review including participants with Bulimia Nervosa. The authors found that individuals with Bulimia tended to report greater embodiment levels compared to controls, even when controlling for mood for embodiment levels as measured by questionnaires. No differences instead were detected when considering proprioceptive drift.

The Full Body Illusion [66] can be seen as an advanced version of the Rubber Hand Illusion. The FBI is a perceptual illusion that, after synchronous visuo-tactile stimulation of the virtual and real bodies, induces individuals to experience a virtual body as their own, similar to the RHI. The main difference between these two procedures is that the first induces embodiment over the whole body, whereas the second induces embodiment over a specific part of the body. Consequently, the site of multisensory stimulation to promote body ownership is different in the two procedures: the FBI requires stimulation of the abdominal area, whereas the RHI requires stimulation of the back of the hand. In addition, the FBI has largely been presented in virtual reality, as it allows the individual to be fully immersed in a virtual environment. The strength of the full-body illusion is measured by self-report measures such as the Embodiment Questionnaire [50] and physiological measures such as skin temperature (e.g., a drop in temperature indicates a stronger illusory experience; [63]. Additionally, Visual Analog Scales (VAS) can be used to assess the emotional activation induced by the experience [63].

Two studies in this review [61, 63] employed Full Body Illusion in Virtual Reality to study body experience in patients with AN. In contrast to previous results with the rubber hand illusion, both studies found that people with AN did not experience the illusion differently than controls, as assessed by skin temperature [63], questionnaires, and VAS [61, 63].

Provenzano et al. [63] presented participants with an additional task: the avatar selection task. Here, participants had to select their ideal and real body shapes from several possible bodies in order to assess body satisfaction, i.e., the differences between the two selected bodies, and perceptual accuracy, i.e., the ability to identify the body most similar to their own among the possible options. While the embodiment procedure was presented from an egocentric frame, i.e., participants looked directly at their stomach to see the virtual body, the avatar selection task was presented from a third-person perspective, i.e., they saw the possible options from an allocentric perspective as objects in front of them. Results showed no significant differences in embodiment levels and no differences in perceptual accuracy, meaning that individuals with Anorexia Nervosa and controls did not differ on all embodiment measures and in the ability to recognize their bodies. Differences emerged instead concerning body satisfaction, where participants with Anorexia Nervosa showed greater levels of dissatisfaction than controls. Related to these findings, they also observed that patients experienced more positive emotions when exposed to thinner bodies compared to higher BMI bodies, in an opposite trend compared to controls.

#### Visual–haptic–proprioceptive integration

One study included in this review [48] examined visual, haptic, and proprioceptive integration abilities in individuals with Anorexia Nervosa compared to healthy controls. Case et al. [48] proposed participants with the Size-Weight Illusion (SWI; [51], which is an experimental paradigm where two objects equal in shape and mass but different in size have to be compared to determine which one is heavier. The SWI occurs because of multisensory conflicts, under the implicit assumptions that large objects are heavier than small ones and that two objects of equal measure have the same weight [59]. When proposing this procedure to patients with AN, Case, et al. [48] observed a significantly reduced SWI (and reduced “reverse” SWI) in participants with Anorexia Nervosa as compared to controls, despite a baseline normal weight discrimination ability.

The visual, haptic, and proprioceptive integration process in individuals with Bulimia Nervosa was not examined in the included studies.

#### Visual–haptic–proprioceptive–vestibular integration

In the study by Guardia et al. [59], the integration of visual, haptic, proprioceptive, and vestibular information was examined in individuals with AN using the Subjective Vertical (SV) task. The SV task [53] is a measure of spatial orientation constancy that requires manual adjustment of a rod to a perceived vertical position. Spatial orientation constancy is not maintained in healthy individuals under certain circumstances: specific conditions such as darkness or head and/or body tilt lead to systematic deviations in SV [53]. These deviations are referred to as the A and E effects. The A effect is characterized by SV deviations toward the head axis and is typically associated with vision and large tilts, whereas the E effect is characterized by SV deviations away from the head axis and is typically associated with tactile adaptation [59].

In their study, Guardia et al. [59] asked participants with Anorexia Nervosa and healthy controls to make visual and tactile spatial orientation judgments by adjusting a rod toward the vertical in an upright position and with lateral whole-body tilt (90° clockwise or counterclockwise from the vertical line). Results showed that the tactile and visual SV measured in the upright position was very close to the gravitational vertical axis in both groups, whereas differences emerged in the tilted body conditions, where patients showed deviations of the tactile and visual SV with a greater A-effect (i.e., the bar was moved toward the head axis) regardless of the specific side. Notably, no baseline unimodal tactile or visual discrimination ability abnormalities were observed between groups. Similarly, tactile and visual body Z-axis judgments were similar between the groups in the upright body position, and differences emerged in the tilted body conditions, where individuals with AN judged the body as more tilted than it was compared to controls.

The integration of visual, tactile, proprioceptive, and vestibular information in patients with Bulimia Nervosa has not been investigated among the included studies.

#### Auditory-visual integration

The study by Chirico et al. [33] investigated whether multimodal integration of auditory and visual information is impaired in patients with Anorexia Nervosa. Specifically, they used the Sound-Induced Flash Illusion (SIFI) to investigate whether individuals with AN presented an impaired temporal discrimination processing of visual-auditory stimuli compared to healthy controls. The SIFI [55] presents respondents with auditory and visual stimuli at different stimulus onset asynchronies (SOA; 70 ms, 110 ms, 150 ms, 230 ms) and requires participants to determine the number of either visual or auditory stimuli presented in different conditions (only visual, only auditory, and bimodal auditory-visual stimulation). Overall, they found that individuals with AN showed lower accuracy compared to HCs for each SOA and presentation modality. Moreover, patients tended to report more incorrect responses compared to healthy controls. The only exception was found for a shorter interval between the onset of two visual stimuli (from 70 to 110 ms), where there were no significant differences between the groups. However, impaired performance at longer SOA was reported by the former in the visual condition (150 ms, 230 ms). Notably, individuals with AN were never able to detect the double tone in the auditory condition. Instead, they reported a higher number of correct responses in the visual condition compared to the other two modalities. Abnormalities in patients with Anorexia Nervosa compared to controls were observed for longer visual SOA, as well as for the bimodal condition across all stimulus onset asynchronies, with a shorter temporal binding window (i.e., the time interval in which stimuli are perceived as occurring simultaneously).

None of the included studies investigated multimodal integration of visual, tactile, and proprioceptive information in individuals with Bulimia Nervosa.

#### Visuo-tactile integration

The study by Risso et al. [64] assessed multisensory deficits in AN focusing on how participants integrate crossmodal sensory information. For this purpose, the Multisensory Processing Assessment Task (MPA; [54]) was used. The MPA allows the assessment of sensory and multisensory processing, taking into account the reliability of both visual and tactile unisensory modalities as well as their integration. The task requires participants to discriminate the shape of small ellipses using only visual, tactile, or both visual and tactile information. The experimenter places a stimulus plate with a raised ellipse on it in the structure and positions it behind a hole in the plate. In the visual condition, participants see the raised ellipse through a hole in the plate, whereas in the tactile condition, they have to move their arms behind the plate and feel the raised ellipse without being able to see it. In contrast, in the bimodal conditions, participants simultaneously see and touch a double-sided printed ellipse, seeing the ellipse on the front of the stimulus panel while simultaneously touching the ellipse on the back. Concerning the shape distortion of the ellipses, Risso et al. [64] found that participants with Anorexia Nervosa tended to overestimate the ellipses more than healthy controls in the tactile modality, whereas no differences emerged in the visual domain, where the level of accuracy was relatively high in both groups. Discrimination thresholds showed that patients had higher visual and tactile thresholds than controls. Analysis of multimodal integration in the bimodal conditions revealed that patients with AN integrated tactile and visual information as well as controls in terms of accuracy, but differences emerged in terms of discrimination thresholds, with patients showing a lower bimodal threshold than unimodal, whereas healthy controls reported no significantly different thresholds across conditions.

The integration of visual and tactile information in individuals with Bulimia Nervosa was not explored in any of the included studies.

## Discussion

This systematic review of the literature identified thirteen studies investigating multisensory integration abilities in patients affected by Anorexia Nervosa, of which only one study included individuals with Bulimia Nervosa [57]. No studies were found that examined brain function during multisensory integration tasks in these conditions.

Overall, behavioral studies provide evidence for alterations in Visual–Haptic–Proprioceptive–Vestibular [59], Visual–Haptic–Proprioceptive [48], Auditory–Visual [33], and Visual–Proprioceptive–Vestibular integration [42, 56, 59, 60, 62] in individuals with Anorexia Nervosa. Concerns arise when considering visuo–tactile integration [64] and visual-proprioceptive-vestibular integration, where different paradigms have yielded inconsistent results. Specifically, in this regard, patients with AN showed an abnormal illusory experience during the Rubber Hand Illusion [52, 57], but contrasted results were found when using the Full Body Illusion [61, 63]. The small number of studies included in this review suggests that multimodal integration processing has received little attention in Anorexia Nervosa, despite available evidence suggesting possible abnormalities in this process [16]. In contrast, there has been minimal research on Bulimia Nervosa. Therefore, future studies are needed to clarify possible differences and similarities between the two conditions to understand how patients affected by these pathologies experience their bodies.

In the following sections, we will discuss the results of the studies according to the sensory modalities involved, attempting to integrate the behavioral results of this review with neuropsychological and neuroscientific evidence.

### Visual–proprioceptive–vestibular integration

Findings from this review [42, 56, 58, 60, 62] suggest the presence of alterations in the integration of visual-proprioceptive-vestibular information integration in patients with Anorexia Nervosa. Indeed, all included studies found that individuals with AN tended to report an abnormally higher critical aperture-to-shoulder ratio compared to healthy controls in body-scaled action tasks [46].

One possible explanation for this difference may rely on distorted body information stored in memory: if the body representation stored in memory is inaccurate, it may mislead both perceptual and action-related body representations. That is, patients may process and program motor responses accurately, but based on altered body size information retrieved from memory [67]. In addition, negative emotional top-down processes may also be involved: individuals with AN indeed show high levels of negative affect (e.g., body dissatisfaction), and such emotional aspects have been found to influence both motor decisions and size estimations [67]. Although these aspects were not investigated in the studies included in this review, they may partially account for their findings. Another possible explanation for the motor task findings may be related to locomotor variables, such as walking speed [68]. For example, it may be that patients tend to have a faster walking speed during the task, which in turn may lead to less accuracy and attention during the action, resulting in less accurate judgments.

Neuroscientific studies may also partially explain the differences observed between patients with Anorexia Nervosa and healthy individuals. Alterations in body schema were found to be associated with lesions in the parietal and dorsolateral frontal cortices [69], and abnormalities in the frontoparietal-cingulate network and the frontal gyrus seemed to be associated with distorted body experience and disturbances in self-identity [70]. Resting-state neurofunctional and neurostructural studies revealed alterations in frontal regions in patients with AN. For example, decreased gray matter volume was observed in the superior frontal gyrus and right middle frontal gyrus [71] as well as at the level of the dorsal and rostral anterior and cingulate cortices [72]. Since body-scale action tasks require movement, another possible explanation for these findings could involve the cerebellum. Indeed, this structure is involved in the integration of information from sensory cortices to ensure sensorimotor coordination [73, 74] and optimal information processing [75]. Some research showed that cerebellar changes are associated with body-scale motor tasks [75], and some neuroimaging studies have highlighted possible structural changes in this region in individuals with AN [71].

The reasons for such cortical differences are still in doubt, but they seem to be related to prolonged food deprivation [76], which might cause deficits in perceptual organization (e.g., the ability to group visual elements to process a visual stimulus as a whole; [77]). If this is true, there should be a relationship between cortical alterations and disease severity (e.g., greater disease severity, greater cortical changes, and MSI deficits). However, data are still limited and results are only partially consistent: additional neuroimaging studies while participants perform multisensory tasks are needed to test this hypothesis. Therefore, we can only conclude that differences in cortical functioning in fronto-parietal regions and the cerebellum may underlie differences between patients with Anorexia Nervosa and healthy individuals in multisensory integration performance as reflected by motor tasks.

### Visual–tactile–proprioceptive integration

In terms of Visual–Tactile–Proprioceptive integration, the studies in this review that used the Rubber Hand Illusion in a sample of individuals with AN [52, 57] found that patients reported significantly greater embodiment over the rubber hand than healthy controls, suggesting an abnormal cross-modal integration process. These findings are consistent with other research showing that individuals affected by Eating Disorders tended to report higher embodiment during the Rubber Hand Illusion than controls, both behaviorally (i.e., proprioceptive drift) and cognitively [78, 79]. Such findings have been interpreted as evidence that patients with AN have a highly malleable bodily self [57]. The authors interpreted their findings as suggesting that patients with Eating Disorders might have a flexible and malleable body experience [52, 57].

One of the reasons for these results may be a tendency of patients to rely on visual inputs in constructing the representation of their own body compared to other sources of information (e.g., proprioceptive, tactile). This was supported by the significant differences in the body illusion regardless of the condition (synchronous or asynchronous). In addition, another possible reason for these findings could be the strong reliance on visual information and the associated difficulty in detecting internal body information (e.g., interoceptive, vestibular, and proprioceptive signals; [21]).

The use of body illusion paradigms as a treatment for distorted body representation in individuals with Eating Disorders is also consistent with this hypothesis: some authors [52, 57] have suggested that the effectiveness of paradigms such as the FBI in modifying body representations may be related precisely to patients' tendency to base their representations on the most recent visual input [56]. This implies that the suggestion of a body other than one's own (i.e., the avatar) would provide visual information capable of modifying body perception, at least temporarily.

The research group led by Eshkevari [80] examined also a sample of patients discharged from the Eating Disorders, including patients who recovered from AN. They found that participants who recovered from Anorexia Nervosa showed significantly higher embodiment scores compared to healthy individuals, but no significant differences emerged concerning proprioceptive drift. Thus, they found a pattern similar to that observed between controls and participants with Bulimia Nervosa [57].

Thus, flexibility in bodily experience seems to persist even after recovery from pathology at the explicit but not implicit level. Several studies have shown the persistence of interoceptive deficits even after recovery from Anorexia Nervosa [81], which could then explain the outcome in terms of a persistent tendency to base one's bodily experience on visual information. However, this tendency may be reduced after recovery, resulting in an inconsistency between explicit and implicit embodiment measures. Interestingly, the similarity between individuals recovered from Anorexia Nervosa and patients with Bulimia Nervosa is consistent with the diagnostic crossover that often leads to an oscillation between these two disorders [82].

A second explanation for why patients with AN embody the rubber hand more than controls may be related to a self-objectification process [9, 83]. Patients who tend to view their bodies as objects may choose to use a visual imagery strategy that emphasizes a third-person perspective, as opposed to a motor strategy that emphasizes a first-person perspective. Indeed, as discussed in the Introduction, self-objectification was the largest contributor to both the onset and maintenance of EDs in two 4-year longitudinal studies [7, 8]. In addition, previous research has found a positive association between embodiment in the RHI illusion and levels of self-objectification [57]. Furthermore, self-objectification may also explain the tendency to prioritize visual information over other bodily signals, as well as negatively affect cognitive functions (e.g., critical thinking, and problem-solving; [84]) necessary to process incoming information.

Neuroscientific studies have found cortical alterations in patients with AN that may account for the behavioral results observed during the RHI. Functional Magnetic Resonance (fMRI) studies showed that the RHI experience is associated with increased activation in the premotor cortices, intraparietal cortices, and cerebellum, suggesting that these regions may be actively involved in resolving multisensory conflicts during body illusions [78]. Furthermore, the precuneus and parietal regions are thought to be particularly involved in the cross-modal integration required for body experience and body illusions [13], and they appear to be involved in the egocentric and allocentric coding of spatial information too [13]. In support of this, previous studies have found alterations in these regions in patients affected by body awareness disorders [76]. Patients with AN generally show lower baseline functional connectivity in all of these areas [71, 85] and research appears to be highly consistent regarding the presence of neurostructural and neurofunctional alterations in the precuneus and parietal areas in patients with AN [86, 87]. Thus, cortical abnormalities at the level of the precuneus and parietal networks may, in part, account for abnormal embodiment and body-ownership experiences during RHI.

Notably, while research has shown alterations in parietal regions and the precuneus in AN, no structural or functional abnormalities have been found in patients with BN [72]. Previous research on BN found a lower degree of cortical alterations in this pathology compared to AN [88]: thus, cortical differences may support the differences in RHI observed between individuals with AN and BN in the study by Eshkevari et al. [57].

Despite this evidence, it is important to note that neuroscientific data regarding cortical impairments in patients with Anorexia Nervosa—as well as Eating Disorders in general—are still contradictory and limited, so it is not possible to find a direct and precise link between brain function and body illusion experience. Indeed, general and broader impairments within brain networks due to both genetic and disordered eating symptomatology (e.g., starvation) may account for behavioral outcomes.

However, the results of this review regarding the integration between visual, tactile, and proprioceptive stimuli are controversial. When assessing cross-modal integration of the same sensory modalities, Provenzano et al. [63] and Keizer et al. [61] observed opposite results: they found that individuals with AN did not report differences in embodiment compared to healthy controls, either at the explicit or implicit level. Instead, they reported significant differences in body satisfaction. These findings seem to support the idea that individuals with AN are characterized by abnormalities in cognitive-emotional rather than perceptual body components.

Through this systematic review, we identified differences between the Rubber Hand and Full Body Illusions, specifically concerning the body site being stimulated to promote the illusion (palm and abdomen, respectively). These two areas differ significantly in emotional valence for patients (the abdomen being more emotionally engaging compared to the hand palm). Thus, affective factors may influence body ownership, as demonstrated by studies in which differences in embodiment levels between groups decreased when controlling for mood [57].

Another potential mechanism involved in such differences concerns cardiovascular and thermoregulatory abnormalities. Previous studies have shown that varying hand temperature and arm blood flow increase the amount of proprioceptive drift during synchronous visuo-tactile stimulation [89]: as Anorexia Nervosa is associated with cardiovascular and thermoregulatory abnormalities, individual differences in these processes may also affect body illusion results reflected by skin temperature [90].

The results by Provenzano et al. [63] highlight the potential effect of the spatial frame of reference in which the tasks were presented. Indeed, the stimulus to induce embodiment over the fake body was presented from a first-person perspective, whereas the ideal and real body choice task was presented from a third-person perspective. Thus, as suggested by the authors, the discrepancies between these two tasks could be interpreted as evidence that patients with AN could be characterized by an impairment in multisensory integration, defined as the ability to combine egocentric and allocentric bodily information [35, 91].

These data are consistent with the proposal offered by the Allocentri lock Theory (ALT; [14, 14, 36, 91]) regarding the aetiology of Eating Disorders. The ALT suggests that patients affected by EDs may have deficits in the ability to integrate allocentric (somatic information stored in memory) and egocentric (incoming sensory information) information through a multisensory integration process [92]. Several studies support the ALT hypothesis, suggesting the use of body illusions to modify the allocentric body of patients affected by Eating Disorders (for more, see [31, 66, 93]). In addition, the ALT found its strength in evidence from neuroscientific studies, specifically focusing on parietal and temporal structures. We have discussed the role of the parietal lobe in shaping body experience in previous sections. The temporal lobe, on the other hand, is involved in visual and auditory processing, while the hippocampus and surrounding temporal structures are involved in long-term spatial memory and the generation of allocentric representations, including bodily ones [94]. Since body experience results from the integration of egocentric and allocentric bodily information [36, 95], alterations in parietal and temporal areas observed in individuals with ED may lead to deficits in the ability to correctly store body-related information from a third-person perspective in memory, resulting in an altered experience of one's own body [94].

### Visual–haptic–proprioceptive integration

Case et al. [48] used Size Weight Illusion to investigate visual-haptic-proprioceptive integration in patients with AN compared to healthy controls, providing evidence for cross-modal sensory integration deficits in individuals with AN. In particular, the reduced sensitivity to the size-weight illusion in patients with AN showed some similarities to the performance of patients with left temporal parietal lesions [96]. As previously discussed, patients with Anorexia Nervosa show widespread cortical alterations, including within temporal and parietal networks [85]. Thus, possible abnormalities in temporal areas may partially support the altered illusory experience in AN.

However, the study by Case et al. [48] has some methodological problems. Indeed, it is not clear which specific modalities are involved (e.g., whether impairments involve visual-proprioceptive or tactile-proprioceptive integration). Other authors identified this limitation, criticizing the inability of Case’s paradigm to distinguish between the contribution of visual and haptic information and to assess the integration of this information with proprioceptive input [97]. Therefore, recent research has proposed an extended version of the Size Weight Illusion by including visual and haptic conditions [97]. Using this version of the task, no differences were found between participants with AN and healthy controls, suggesting no impairment of visual-haptic integration. Thus, multisensory integration difficulties may arise when proprioceptive information needs to be combined with visual and tactile inputs. This hypothesis could be supported by specific nerve fibers involved in the transmission of somatosensory information: indeed, patients with AN show atrophy of type II nerve fibers, which could reduce somatosensory information and lead to an abnormal response to this type of stimuli [89].

### Visual–haptic–proprioceptive–vestibular integration

Visual–Haptic–Proprioceptive–Vestibular integration was investigated by Guardia et al. (2013) using the Subjective Vertical task (SV). Authors found alterations in participants with AN compared to controls, with patients reporting deviations of tactile and visual SV toward the body in tilted conditions. Thus, people with AN showed impairments in the constancy of spatial orientation and thus in the ability to integrate multisensory inputs to make correct spatial judgments. Individuals with Anorexia Nervosa seem to show widespread cortical alterations, including within temporal and parietal networks, which play a critical role in spatial judgments and cross-modal sensory integration [92, 98]. Thus, SV task performance may be associated with changes in brain function.

Furthermore, the increased A-effect in patients with AN might be framed according to the Allocentric Lock Hypothesis [14, 14, 36, 91]. In these terms, patients may be more likely to rely on an allocentric frame of reference due to difficulties in combining egocentric and allocentric information [36]. Also in this context, behavioral data could be related to the atrophy of type II nerve fibers, which could reduce somatosensory information flow and processing [59, 89].

### Auditory–visual integration

Auditory**–**visual integration in individuals with AN is a relatively unexplored area of research. Indeed, only one study in this review investigated this integration process in patients with Anorexia Nervosa, using the Sound-Induced Flash Illusion task [33]. The results of this study indicated that individuals with AN tended to make fewer correct responses than controls, particularly for longer SOA. Although no previous research has been conducted on a sample of patients with AN, and it was not possible to make comparisons across studies, this finding seems to provide additional evidence for an impaired cross-modal integration ability in this pathology.

Similar results were found in individuals with Autism Spectrum Disorders. Similar audiovisual tasks have been proposed for individuals with autism as opposed to healthy individuals, revealing difficulties in multisensory processing in the clinical group [99–101]. This abnormality has been proposed to be related to pathology-specific factors, including cortical alterations in specific brain areas for multisensory integration (e.g., parietal lobe), social deficits (e.g., audiovisual integration is required for face-voice association), and sensory overload (e.g., hypersensitivity to sensory stimuli; [99–101]). Notably, these aspects seem to be common to both Autism Spectrum Disorders and Anorexia Nervosa, and studies have shown that autistic traits are likely to be present in individuals who are affected by AN [102]. Thus, the results from this review may be related in some way to specific features that Autism Spectrum Disorders and Anorexia Nervosa have in common.

### Visuo-tactile integration

The visuo-tactile integration process was assessed by only one study included in the review [64]. Here, individuals with AN were presented with the Multisensory Processing Assessment Task (MPA), which assessed both individual visual and tactile sensory modalities as well as the visuo-tactile MSI process. Results showed that patients with AN tended to overestimate stimulus width more than controls, which is consistent with previous research showing that patients with AN overestimate the distance between two points [103]. Since a similar pattern was not found in individuals with high body concern (BCHC), data suggest that this alteration is not related to body concern or other bodily cognitive-affective factors. This supports the idea that patients with AN may show perceptual alterations that are something different compared to body concerns and body dissatisfaction. However, it is not clear whether the higher threshold was due to the precision of the comparison or estimation processes. Based on the results of Risso et al. [64], patients seem to be characterized by unimodal low-level processing deficits in tactile and visual modalities, but not in the integration of such information [103, 104].

However, the interpretation of these results might be misleading because of methodological issues. Indeed, results may be influenced by the way the discrimination threshold was measured, given the small number of trials, as well as by sample characteristics (e.g., disease duration which may affect the severity of multisensory abilities) that were not extensively collected and/or reported. Given these concerns, the results should be interpreted with caution.

Neuroscience studies seem to suggest that the presence of visuo-tactile integration alterations in patients with eating disorders may be related to the activity of the somatosensory network [85, 89]. Indeed, the somatosensory system is involved in visuo-tactile integration, among other functions [105], and it contributes to the processing of body-related information across sensory modalities [106] suggesting its role in multisensory integration [105, 107]. Alterations in this circuit could then lead to deficits in integrating visual-tactile stimuli, as supported by the affective-touch literature [108]. However, the data in this review do not support this hypothesis. A possible reason for this discrepancy may be related to emotional factors: while visuotactile deficits may be observed when affective touch stimuli are used, no deficits may be found when emotionally neutral stimuli are presented. Thus, cross-modal integration involving touch may be significantly influenced by social and emotional factors more than other sensory domains [109]. This is similar to what has been previously discussed regarding Visual–Tactile–Proprioceptive integration. Consistent with this proposal, previous studies have shown that high levels of body dissatisfaction are associated with inaccuracy in tactile distance estimation (e.g., [103]), stressing a significant influence of interpersonal and affective factors in tactile processing.

### Summary of findings

In this systematic review, we aimed to outline the findings of studies on individuals with Anorexia Nervosa and Bulimia Nervosa using multisensory tasks to investigate multimodal integration abilities in these conditions. Based on the available evidence, individuals with AN appear to exhibit abnormalities in the ability to combine inputs from different sensory domains, both in response to body-related (e.g., [63] and non-body-related stimuli (e.g., [33]). The limited evidence available suggests that these changes persist when information from different spatial frames is combined.

Thus, the studies included in this review suggest that AN may be associated with an inability (or suboptimal ability) to integrate sensory information from multiple sensory domains into a unique and coherent percept, which is a core process in shaping bodily experience. Deficits appear to occur particularly when internal body signals (i.e., proprioceptive, vestibular) must be combined with exteroceptive information (i.e., visual). On the other hand, only limited data have been found on patients with BN, so it is not possible to draw conclusions and make comparisons between the two pathologies.

### Limitations

The current review has some limitations that need to be highlighted. First, the limited number of included studies. Only thirteen studies met our inclusion criteria, demonstrating the scarcity of research in this area and the lack of studies focusing on the assessment of MSI abilities in AN and BN. We found that most of the research focused on subclinical or nonclinical conditions and studies that attempted to use multisensory stimulation as a treatment for the somatic affective components (e.g., [110–112]). In addition, only one of the included studies considered patients with BN, which does not allow us to make comparisons and draw conclusions about this pathology. Regarding the sample, not all included studies reported potentially relevant sample characteristics such as disease severity, disease duration, diagnostic subtype, or other clinically relevant variables (e.g., comorbidities or medications). Given the paucity of studies, we did not specifically analyze such confounders, which may limit our interpretation.

Further research should better address the role of these critical factors: for example, it may be that greater EDs severity is associated with greater MSI deficits. This hypothesis is supported by studies conducted in subclinical samples: studies have found a relationship between multisensory integration skills, body image, and eating disorder symptoms, with greater deficits being positively associated with greater symptomatology [44, 113]. In this vein, it may also be the case that deficits in multimodal integration may predict the development of body image-related disorders and individualize individuals at risk for eating disorders. This research will be essential for proposing preventive and early interventions [114]. In addition, further research is needed to understand how the specific clinical subtype (e.g., restricting, binge/purge subtypes of Anorexia Nervosa) affects multimodal integration ability.

Another striking argument relates to gender differences: most of the studies included in this review focus on female patients. Since Eating Disorders are also increasing in males [115], further research should include male patients to examine gender differences, thus comparing not only male patients with healthy controls but also females with male patients with the same diagnosis.

Finally, we did not find any neuroimaging and neurofunctional studies that included multimodal tasks: this limited us to hypothesizing and speculating the neurobiological basis of multisensory integration without the possibility of anchoring behavioral findings to robust neuroscientific results. Thus, we encourage future studies to use fMRI techniques to investigate cortical activations during cross-modal tasks to better reveal cortical functional differences between patients and controls.

## Conclusions and further directions

To our knowledge, this systematic review is the first attempt to systematically investigate the multisensory integration abilities in patients affected by Anorexia Nervosa and Bulimia Nervosa. Crossmodal integration refers to the combination of sensory information from different sensory modalities (e.g., tactile, visual, auditory, proprioceptive, vestibular) and spatial reference frames (egocentric and allocentric) into a unique and coherent percept [24, 26], and constitutes a crucial process for body perception [47].

Body representation and image disturbances have long been recognized as critical factors in the development and maintenance of eating disorders, specifically in Anorexia Nervosa and Bulimia Nervosa [116]. Indeed, a large body of evidence has demonstrated that patients consistently exhibit disturbances in the way they feel, think, and perceive their bodies. While cognitive and affective body components have been extensively investigated in the literature, the perceptual aspect has received less attention. Therefore, in this systematic review, we summarize the evidence on bodily experience in these conditions by looking at its most fundamental process, multisensory integration. We argue that this is necessary to fully understand the body experience of people with these conditions. The studies included in this review revealed that individuals with Anorexia Nervosa show an inability (or less than ideal ability) to combine multimodal sensory information and information from different spatial frames into a single and coherent percept.

Further research is needed to understand the reasons for the in-depth understanding of multimodal integration abnormalities. Previous research has shown that patients with AN have deficits in the processing of interoceptive signals (see [21]), whereas no impairments were found when processing unisensory exteroceptive information [117]. This alone could explain the MSI difficulties, as interoceptive information is still biased before integration takes place [118]. Additionally, cortical alterations in regions involved in MSI (i.e., frontal, parietal, temporal areas) might partially account for those results too [104]. However, we argue that single-level accounts of MSI alterations (e.g., only biological factors) are too reductionist on their own and will only reach their full value when embedded in a more complex, multi-level explanatory framework that can account for the influence of both bottom-up and top-down processes.

**Fig. 3:**
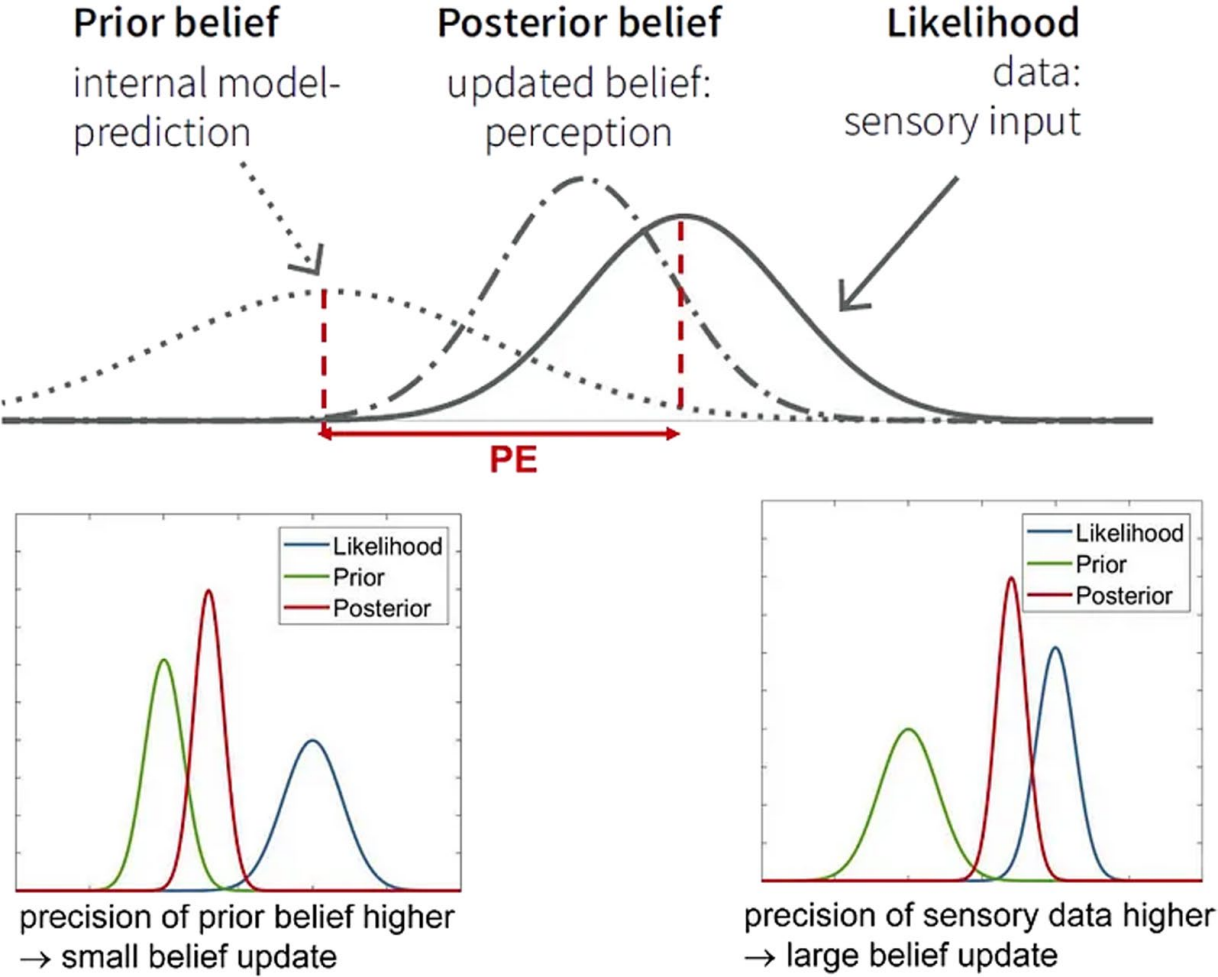
The outcome of the predictive coding process depends on weight (precision) attribution. On the left: when priors are judged to be more precise than incoming sensory information (likelihood), the posterior will be primarily based on priors (i.e., the posterior is shifted towards priors and there is no updating of existing beliefs); on the right: if the likelihood is judged to be more accurate than priors, the posterior will be mainly based on incoming sensory information (i.e., the posterior is shifted toward priors and there is belief update; prior blinding; image from [122])

We, therefore, propose the Predictive Coding (PP; [119]) framework for understanding MSI abnormalities in ED. PP views the brain as a "Bayesian prediction machine" that actively constructs percepts. The basic idea is that the brain compares incoming sensory information (likelihood) with internal representations (internal models) based on prior knowledge and beliefs (priors) through an active and iterative process. The discrepancy between expectations and actual sensory data defines the prediction error (PE). According to the “free energy” principle, the PE must be minimized, and this can be done by updating predictions (i.e., adjusting the prediction based on incoming information) or by active inference (i.e., acting to adapt incoming information to predictions; [119]. The strategy adopted depends on the accuracy and reliability (precision) assigned to priors and incoming information: new signals can adjust priors and vice versa, depending on which source is considered the most precise (Fig. 3). It follows that inappropriate precision weighting (e.g., giving too much or too little weight to either the prior or the likelihood) affects PE gain and the ability to update internal models, leading to erroneous inferences [104, 120]. When sensory information is weighted as unreliable, the final output (posterior) is primarily based on priors, in what has been termed the “prior tendency to bind stimuli” [120]. This happens for instance when sensory data are noisy, but also as a result of attentional, emotional, and biological factors [121].

In terms of neural implementation, precision, and PE depend on neuromodulators such as acetylcholine, GABA, dopamine, and glutamine [104, 123]. Acetylcholine suppresses PE and regulates its precision, whereas GABA and dopamine determine the influence of PE on the internal model and PE reward respectively; finally, Glutamatergic-*N*-methyl-d-aspartate receptors (NMDAR) send predictive signals from higher hierarchical levels to lower levels [104, 123]. Disruptions in these neuromodulators have been proposed to explain alterations in the inference process in conditions such as psychosis and posttraumatic disorder [104, 124].

**Fig. 4:**
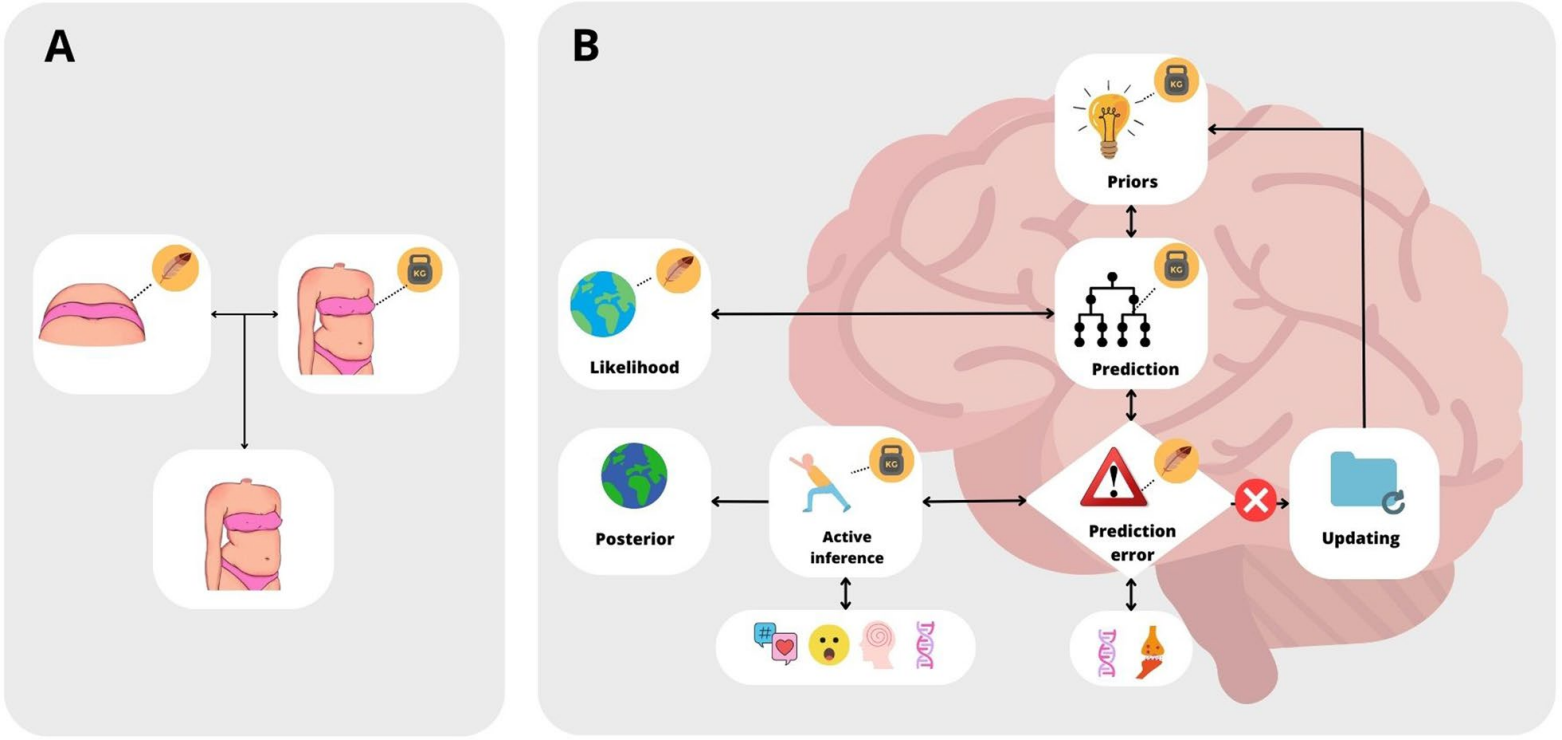
Impaired predictive coding in the context of dual-level multisensory integration. Panel A shows how, when first- and third-person spatial frame signals have to be combined, the integration result is biased towards the latter if a higher level of precision (more weight) is assigned to the allocentric information. Panel B summarizes the critical steps of inference processing when combining information from different sensory domains; changes could occur if more weight is given to priors and predictions than to incoming information, partly due to neurobiological factors. The discrepancy (prediction error) would then be resolved by seeking confirmation of one's predictions through active inference, rather than by updating one's internal models. In this active inference, socio-cultural, emotional, genetic, and neurobiological factors can guide the search for and processing of the information encountered. Therefore, the result (posterior) will be biased and not fully consistent with the original incoming sensory information

Alterations in NMDAR receptors [123, 125], as well as the neuromodulators GABA, [126], dopamine, [127], and acetylcholine [128, 129], have been reported in individuals with AN. This imbalance could lead to an altered precision weighting process, resulting in much more weight being given to prior beliefs than to incoming information, and therefore the PE will be considered imprecise (i.e., insufficient to update the internal model). Thus, the MSI posterior will be biased toward priors rather than sensory information, and the PE signaling the discrepancy between the two will not be strong enough to update the internal model. In this sense, individuals with AN may be characterized by an abnormal "prior blinding" tendency. Other factors might also contribute to the bias in the process, such as altered interoceptive signal processing [118], the different precision attributed to each sensory modality (e.g., visual information might be considered more reliable than others; [114]) as well as biological factors (i.e., genetic and neurobiological changes affecting information processing; [130]). The resulting weak PE will be thus solved through active inference, where sociocultural and psychological factors may strengthen the internal model (Fig. 4).

This prior blinding effect has negative consequences when priors are distorted. Patients with EDs do indeed hold maladaptive beliefs (e.g., the ideal of thinness), and neuroscience studies have shown cortical dysfunction in memory-related cortical areas (e.g., distorted body representation in memory; [131, 132]. As suggested by a systematic review of resting-state functional-MRI studies [34] “several brain regions could be involved in body image disturbances and may sustain an impaired integration between real and perceived internal/external state of one's own body in AN patients” (p. 582). Thus, basing body perception on dysfunctional priors might lead to altered body experience.

In particular, the final posterior requires the integration of information from both egocentric and allocentric spatial frames. The same altered processing could affect both egocentric and allocentric processing. Moreover, when there is spatiotemporal synchrony between information coming from different frames, the information is combined based on the weight of each submodel [133] A VR study by Serino et al. [134] suggests the existence of a primary impairment in the processing of spatial reference frames in patients with EDs, which in turn might alter the final inferential output.

Self-objectification might lead individuals with Eating Disorders to consider allocentric information as more precise than egocentric information, so that all signals are aligned with the dominant frame [28] and to weigh visual information as more reliable compared to other senses. Information as more reliable compared to other senses. Furthermore, as shown by Eich et al. [135], the use of an allocentric perspective turns off interoceptive signals. Commenting on their results, the authors explain: “The data suggest that adopting an observer [allocentric] perspective is tantamount to literal disembodiment at the neural level. That is, when we choose to relive past events from a perspective outside our body, we shut down the neural circuitry in the insula that is central for monitoring our bodies’ internal states.” (p. 177). As the Allocentric Lock theory suggests [12, 35, 95, 136], people with AN may live in an "allocentric" body in which priors (e.g., beliefs, expectations, memories) have an enormous influence on shaping their experience relative to new incoming sensory information, including interoceptive information. As recently demonstrated, individuals with AN favor interoceptive metacognitive processes (e.g., trusting their own perceived sensations rather than their actual perceptions), disregarding bottom-up bodily input in favor of their previously altered top-down beliefs [137]. In other words, they experience the wrong body that they expect to experience.

Existing models of EDs tend to underestimate the role of body experience in the development and maintenance of pathology [136] although, as discussed in the Introduction, the results of 4-year longitudinal studies involving more than 5,000 individuals have underscored its importance [7, 8].

Based on the data from this review, we suggest that the MSI process may be a critical mechanism in eating disorders and that its interaction with other factors (biological, sociocultural, psychological; [138, 139]) may play a critical role in shaping the pathological outcome. In this framework, PP can be used to predict and understand how all these elements interact to determine a phenomenological outcome with specific characteristics, taking into account individual differences. However, the small number of studies encourages further research to investigate the possible relationship between multisensory integration and eating disorders. This can be done, for example, using multisensory technologies (e.g., VR) and paradigms such as the full-body illusion and the Body Swap illusion [66].

Body illusions use multisensory conflicts to promote embodiment over virtual bodies and offer the possibility of targeting body experience from the bottom up. Virtual reality's ability to simulate predictive processing in the brain and its multisensory nature has made this technology a potential ally in the treatment of body misperceptions in patients with Eating Disorders, particularly Anorexia Nervosa [37, 139]. The underlying idea is that the use of this technology can transform body representation by targeting the underlying mechanisms, i.e. the inferential process in the case of crossmodal integration [31, 61, 66, 93, 129, 140].

To clinically address MSI dysfunctions we recently suggested a new therapeutic approach—Regenerative Virtual Therapy [21, 141]—that integrates VR with different technologies and clinical strategies to regenerate a faulty bodily experience by stimulating the multisensory brain mechanisms combining both exteroceptive and interoceptive stimulation (i.e., visuo-tactile and sonoception respectively;[142, 143]. Furthermore, we propose to integrate the reference frame shift during body illusions, proposing body swapping and/or full body illusions from both egocentric and allocentric spatial frames (i.e., egocentric and allocentric; [144]) to fully target MSI. In this regard, in a recent study, we found that allocentric Full Body Illusion was able to induce embodiment over the fake body (see [144]), altering multimodal integration abilities in healthy individuals: future research should investigate such change in other populations, as well as the impact of allocentric body illusion on factors such as body size estimation, body satisfaction, or body shame.

Data on patients with Bulimia are still too limited to conclude. As previously discussed, we encourage future work to study individuals with BN and to deepen the understanding of this condition to analyze whether multisensory integration deficits are also observed in BN and, if so, whether they have some associations with BN symptoms. This line of research may be relevant not only to better understand BN but also to identify differences and similarities between Anorexia Nervosa and Bulimia Nervosa, as well as why patients tend to transition from one pathology to the other [145].

Although multisensory integration occurs at every moment of our lives, surprisingly few studies have focused specifically on this topic, and several questions remain about this very important computational process in the brain. We propose that the study of MSI may provide a piece of the puzzle to better understand EDs. This does not mean that MSI alone will be able to explain complex conditions such as Eating Disorders, but adding this element to the existing biological and social factors involved in the aetiology of such conditions may offer important opportunities to take a step forward in their understanding.

## Data Availability

All relevant data are within the manuscript and supplementary materials.

## Abbreviations

AN: Anorexia Nervosa
BN: Bulimia Nervosa
VR: Virtual reality
EDs: Eating disorders
ED: Eating disorder
HCs: Healthy controls
BMI: Body mass index
MSI: Multisensory integration processing
RHI: Rubber hand illusion
FBI: Full body illusion
cA/S: Critical aperture to shoulder ratio
SWI: Size weight illusion
SIFI: Sound- induced flash illusion
SV: Subjective vertical task
MPA: Multisensory processsing assessment task
fMRI: Functional magnetic resonance
PP: Predictive coding
PE: Prediction error
F: Female
ENDOS: Eating disorder not otherwise specified
BCHC: Healthy controls with high body image concerns
NMDAR: Glutamatergic-*N*-methyl-d-aspartate receptors
GABA: Gamma-aminobutyric acid
SOA: Stimulus onsent asynchrony
SD: Standard deviation
DSM IV: Diagnostic and statistical manual of mental disorders fourth edition
DSM-IV-TR: Diagnostic and statistical manual of mental disorders, 4th edition, text revision
DSM 5: Diagnostic and statistical manual of mental disorders fifth edition
ICD-10: International classification of diseases, tenth revision
